# Quantifying stochastic establishment of mutants in microbial adaptation

**DOI:** 10.1099/mic.0.001365

**Published:** 2023-08-10

**Authors:** Helen K. Alexander

**Affiliations:** ^1^​ Institute of Ecology & Evolution, University of Edinburgh, Edinburgh, UK

**Keywords:** demographic stochasticity, establishment probability, experimental evolution, extinction, fixation probability, gene surfing, genetic drift, mutation, population dynamics, predictability, seeding assay, statistical inference

## Abstract

Studies of microbial evolution, especially in applied contexts, have focused on the role of selection in shaping predictable, adaptive responses to the environment. However, chance events – the appearance of novel genetic variants and their *establishment*, i.e. outgrowth from a single cell to a sizeable population – also play critical initiating roles in adaptation. Stochasticity in establishment has received little attention in microbiology, potentially due to lack of awareness as well as practical challenges in quantification. However, methods for high-replicate culturing, mutant labelling and detection, and statistical inference now make it feasible to experimentally quantify the establishment probability of specific adaptive genotypes. I review methods that have emerged over the past decade, including experimental design and mathematical formulas to estimate establishment probability from data. Quantifying establishment in further biological settings and comparing empirical estimates to theoretical predictions represent exciting future directions. More broadly, recognition that adaptive genotypes may be stochastically lost while rare is significant both for interpreting common lab assays and for designing interventions to promote or inhibit microbial evolution.

## Data Summary

A text description and R scripts for simulating two simple models (I. The stochastic birth-death model of cell population dynamics; II. Fluctuation assays with stochastic establishment of mutants) are deposited on FigShare [1]: 10.6084 /m9.figshare.23601711

## Background

Experimental evolution of microbes is now a widespread and powerful technique both to test general evolutionary theory and to generate microbial adaptations of applied interest [2, 3]. Further investigations can identify phenotypic and genotypic changes underlying adaptation, and conversely, environmental conditions under which particular changes are adaptive – for instance, antimicrobial concentrations that select for resistance [4, 5]. Studying microbes primarily in bulk culture, in vast population sizes, has promoted a focus on the predictable force of selection. However, no matter how large the population, *de novo* genetic changes (mutation or horizontal gene transfer [HGT]) initially arise in single individuals. This introduces a fundamental layer of stochasticity, or randomness, into adaptation [6].

First, as long recognized by microbiologists, appearance of specific adaptive mutations is rare and hence highly variable. Luria and Delbrück famously exploited the pattern of variation in mutant numbers across replicate cultures to draw a fundamental conclusion in evolutionary biology, that mutations arise at random [7]. Their experiment, now known as a fluctuation assay, has become a common method of estimating mutation rate that accounts for stochasticity [8–10].

**Fig. 1.**
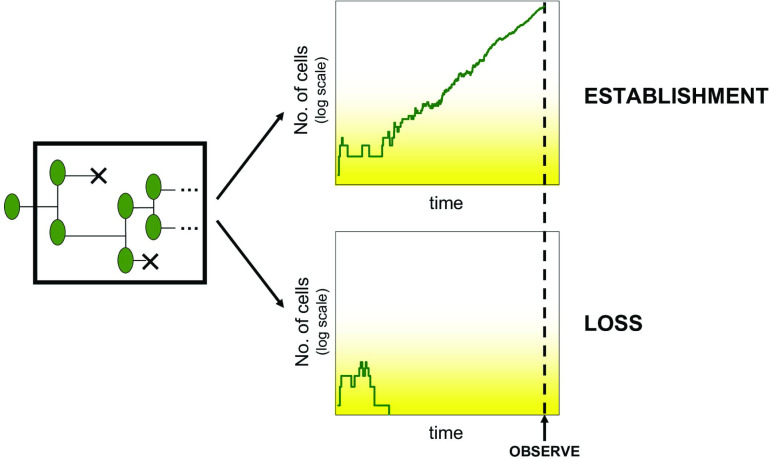
Conceptual view of establishment. A lineage initiated by a single cell undergoes replication and death events. Stochasticity in these events leads either to establishment or loss of the lineage. The influence of stochasticity is strongest at small population sizes and reduces as population size increases (fading-out yellow shading). Experimentally, we usually do not observe underlying demographic dynamics (black box), but only the final outcome at the observation time. Detection of a sufficiently large population is interpreted as establishment.

Less widely recognized in microbiology, however, is that appearance of mutants does not guarantee outgrowth to levels we detect or care about. Even if a genetic change is selectively favoured and, on average, should grow in numbers, any individual cell could fail to survive or reproduce (variation known as *demographic stochasticity*). If the first mutant cell or its earliest descendants are ‘unlucky’, the entire lineage is lost (Fig. 1). I will use the term *establishment*, in line with many recent authors [6, 11–16], for the event that a mutant lineage escapes early stochastic loss (also known as ‘extinction’ or ‘loss by genetic drift’ [17, 18]) and grows to substantial population size. ‘Substantial’ means the lineage is at negligible risk of subsequent loss by demographic stochasticity alone, and, in practice, is detectable by standard methods (e.g. has formed a visible colony). The mutant need not *fix* (reach 100 % frequency) to be considered established, and in longer-term evolution, might later be out-competed by other genotypes [19].

Stochasticity in the fate of adaptive mutant lineages has important implications for evolution. Although mutations may recur, given enough time in large populations, their timing and order matters. Amongst several possible adaptive mutations, the first to establish can set a population onto a distinct evolutionary trajectory, with subsequent adaptation contingent on earlier changes [2, 20]. Both mutational supply and establishment probability thus affect repeatability of evolution across replicate populations [20, 21]. Moreover, under severe environmental challenges, adaptation may be time-limited: rapid establishment of adaptive alleles is critical for ‘evolutionary rescue’, the avoidance of extinction in declining populations [22]. For example, a pathogen population causing an infection declines under antimicrobial treatment, but can rebound if a drug resistance mutation (or horizontally transferred gene) establishes before the infection is cleared.

The probability of establishment or fixation of a novel beneficial allele is of long-standing interest in theoretical population genetics (reviewed by [23]). (Note that many mathematical models neglect subsequent mutations and competition amongst multiple genotypes, implying that every established beneficial mutation will fix [19].) For instance, in the 1920s, Haldane derived under a simple demographic model that establishment probability is approximately two times the selective advantage of a weakly beneficial mutation arising within a large, stable population [24]. This well-known result predicts that adaptive alleles frequently fail to invade, a qualitative feature recapitulated by more complex models [23]. Even in the absence of competitors, genotypes with positive absolute fitness (expected growth rate) may fail to establish, simply by ‘bad luck’ in survival and reproduction, especially in sub-optimal environmental conditions.

Importantly, conventional growth rate or competition assays are not sufficient to predict establishment probability. The same net population growth (cell divisions minus deaths) can be attained by a strain with either high or low absolute rates of division and death, but a strain with higher mortality is less likely to establish. (These effects can be explored through simulations in the Supplementary Material [1].) Generally, establishment is sensitive to life cycle and the specific step affected by a beneficial mutation [23]. Frequency-dependent or density-dependent fitness could further alter the fate of initially rare mutants compared to larger inocula. Finally, in the case of environmental variation, fluctuating growth rates average out over the long term in large populations, but smaller populations are at greater risk of extinction in transiently poor conditions. Standard fitness measures in large populations fail to capture these effects, and hence a tailored assay is required to estimate establishment probability.

Despite extensive theoretical study, experimental quantification of establishment probability was, until recently, entirely lacking [23]. I suggest that the barriers were both conceptual and methodological. Disciplinary divides likely limited translation of population genetics questions to applied microbiologists. The relevance of demographic stochasticity is not immediately apparent in bulk microbial cultures, where total population size is large and individual lineages are not readily visible. Moreover, stochasticity calls for a shift in mindset: we often equate ‘replication’ with ‘reproducibility’ in microbiology. But rather than running a few replicates to validate a consistent outcome, to study stochastic events we must run many replicates and embrace qualitatively different outcomes (e.g. clearance versus growth). Luria and Delbrück already recognized this issue for mutations, stating that ‘the quantitative study of bacterial variation… has been hampered by the apparent lack of reproducibility of results, which, as we shall show, lies in the very nature of the problem and is an essential element for its analysis’ [7], an insight equally applicable to establishment. However, high experimental replication is laborious and mutant detection is not always straightforward. Analysing stochastic events also requires mathematical and statistical methods that may go beyond many microbiologists’ training. Conversely, theoreticians may lack familiarity with experimental techniques to translate their work into relevant and accessible tools.

I argue that technical barriers to quantifying establishment probability in experimentally tractable microbes have largely been eliminated. Modern culturing tools (micro-titre plates, multi-channel pipettes, lab automation) greatly facilitate high-replicate experiments. Advances in genetic engineering and lab equipment (including plate readers, flow cytometers, and microscopes capable of fluorescence detection) facilitate labelling and detecting mutant strains. Relevant statistical inference methods and code-sharing platforms exist. In parallel, advances in microfluidics and time-lapse microscopy have fuelled an explosion in single-cell-level studies [25], revealing variation that is obscured in bulk culture and further motivating stochastic descriptions for the fate of individual lineages [26].

Over the past decade, a handful of studies have quantified per-cell establishment probability of adaptive mutants in bacteria and fungi [11–15, 17, 27–31]. In this review, I aim to demonstrate both the feasibility and the importance of these approaches, and argue that the time is ripe to expand investigations.

## Methods for estimating establishment probability

**Fig. 2.**
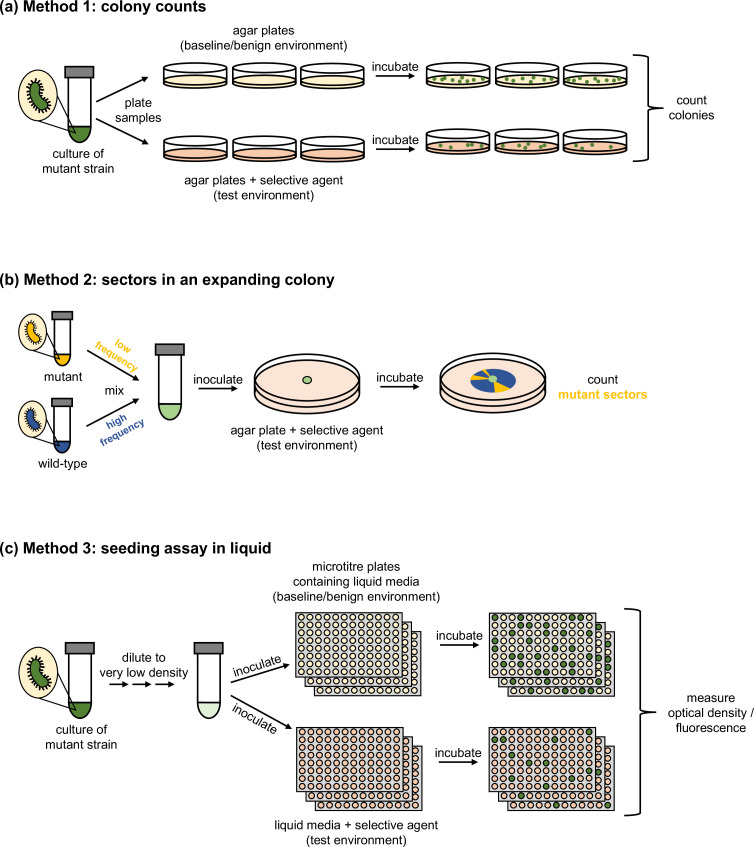
Methods of estimating establishment probability. Panels (**a**)–(**c**) illustrate experimental design for methods 1–3 as described in the main text. In (**a**) and (**c**), only the focal mutant strain is illustrated, but a wild-type strain could be co-inoculated. In (**b**), separate inoculum size estimation is not illustrated. In all cases, multiple ‘test’ environments could be assayed in parallel.

Existing methods to estimate establishment probability of specific genotypes in microbes (Fig. 2) share several commonalities (Box 1). Establishment is assessed by inoculating cells of a known ‘mutant’ strain into an environment of interest, and estimating the proportion of cells that give rise to sufficiently large (i.e. detectable) lineages, typically within one or a few days for fast-growing microbes. Amongst the three existing methods described below, methods 1 and 2 rely on directly counting visible established lineages on solid media, whereas method 3 involves inferring the number of established lineages from outgrowth detected in liquid culture.

Box 1. **Key ingredients for estimating per-cell establishment probability**

- Existing mutant (and optionally wild-type) strain(s) for testing
  - Mutants are previously generated through experimental evolution, random mutagenesis, or genetic engineering
  - Mutant and wild-type normally differ only in the focal beneficial allele and distinguishable markers (e.g. colour, fluorescence or another selectable allele)

- High experimental replication
- Estimate of mutant inoculum size, i.e. how many lineages were initiated
- Method of detecting sufficiently large mutant populations, e.g. visible colonies/sectors or liquid culture turbidity, plus marker detection in co-cultures

There are several nuances in the interpretation of establishment probability estimates (see Box 2 for further details). Most importantly, the working definition of ‘establishment’ depends on detection method and timing: scoring establishment too early or with an insensitive method risks missing lineages that are still growing. We expect the proportion of replicates showing growth to saturate over time, but in absence of a detailed quantitative model, observation time must be chosen pragmatically. The experimenter could observe repeatedly to check that the number of replicates scored as ‘established’ has stabilized. Nonetheless, we cannot entirely preclude surviving lineages that fail to grow above the detection limit within the duration of the experiment. Moreover, if we estimated establishment probability of a given strain using each of the three methods, we would not generally expect to obtain the same results: each approach presents a distinct environment and scores establishment in different ways.

Box 2. **Interpreting establishment probability estimates (‘But what if…?’)**

Q: What if inoculated cells are heterogeneous, such that some are more likely to establish than others?

A: The estimated establishment probability should be interpreted as an average across inoculated cells – i.e. the chance that a randomly chosen cell establishes, or the fraction of cells that establish ([12], Suppl. Text 10.1).

Q: What if a lineage acquires another mutation during the course of its establishment?

A: The chance of this event – or any other demographic, phenotypic, or genetic changes (typically unobserved) that may occur within lineages – is integrated into an overall probability that an inoculated cell produces a detectable lineage.

Q: What if the environment changes over time during the establishment assay (either experimentally imposed or due to microbes modifying their own environment)?

A: Establishment probability is an endpoint measure, reflecting the net impact of conditions over the entire time course of the assay.

Q: What if cells are not guaranteed to establish in the ‘benign’ conditions used to estimate inoculum size, i.e. *p*_e_(0) < 1?

A: The estimated inoculum size can be interpreted as an ‘effective’ value, equal to actual inoculum size times *p*_e_(0), similar to quantifying cell density by colony-forming units. Establishment probability estimates in test environments should then be interpreted as relative chances, i.e. *p*_e_(*x*) should be replaced by *p*_e_(*x*)/*p*_e_(0) in equations 1, 2 and 7. This quantity is not a true probability, and could exceed 1 if a test environment gives higher chances of establishment than the baseline. See [12] for full equations expressed in these terms for method 3.

Q: What if we estimate ‘establishment probability’ larger than 1?

A: The test environment might actually give higher chances of establishment than the baseline (cf. previous *Q*), in which case the choice of baseline might be reconsidered. Alternatively, chances are similar enough to give estimates >1 by sampling error. Quantifying uncertainty should distinguish these possibilities.

Q: What if we estimate different establishment probabilities of the same mutant strain using different methods?

A: These results would not be surprising, because the methods described here present distinct environments, potentially impacting mutant fitness. For example, antibiotic susceptibility is sensitive to environmental factors [60 [Bibr R60]], sometimes even differing between solid and liquid media [61]. Within a dense colony (method 2), mechanical cell-cell and cell-surface interactions affect mutant growth [29, 62]. Finally, even if a mutant lineage grows to the same population size in every case, different detection methods imply it may be scored as established in one method but not another.

In all cases, these methods give empirical, endpoint measures of establishment. This outcome – pragmatically described as ‘stochastic’ – is the net result of potentially complex underlying dynamics. While offering interesting avenues for deeper investigations, these dynamics can be treated as a ‘black box’ for current purposes. That is, we need not observe cell divisions and deaths (see however [30]), nor identify reasons for establishment or loss of individual lineages, in order to estimate the overall probability of establishment.

Finally, note that these methods are agnostic to our motivation for testing a particular genotype, and thus open to alternative interpretations. In an evolutionary context, we conceive of novel genotypes arising by mutation or HGT in a ‘wild-type’ background. Experiments testing establishment can mimic this situation using pre-existing strains, by inoculating mutants at low frequency into a large wild-type population [11–14, 17, 18, 28, 29, 31]. This approach can alternatively been seen as a test of evolutionary rescue from (experimentally controlled) standing genetic variation [32]. Here we consider mutants with a selective advantage, i.e. higher relative fitness than the wild-type. However, even in absence of wild-type competitors, establishment may fail due to the random nature of cell replication and death, especially in presence of abiotic stressors such as antimicrobials. Many studies test the impact of environmental conditions on establishment of a focal genotype in isolation [12, 15, 27, 30, 31], requiring only positive absolute fitness, i.e. average population growth rate, to allow (but not guarantee) establishment. I will still refer to the focal genotype as ‘mutant’ for consistency, and often an adaptive ‘mutation’ of interest (e.g. antimicrobial resistance) is in mind. However, a genetically similar ‘wild-type’ need not exist. These methods can equally well be applied to ecological interpretations of establishment, such as colonization of habitats or infection of hosts following dispersal (e.g. [33–35]), though a full review of these applications is beyond the present scope.

### Method 1: colony counts

The most direct and intuitive way to estimate establishment probability is by counting visible colonies formed when mutant cells are plated and incubated on solid media (agar). We assume that each colony derives from one cell and thus represents one established lineage. With this approach, establishment probability is also called ‘plating efficiency’ [30]. This method has so far been used to test establishment of bacteria at various antibiotic concentrations [15, 27, 30, 31], including cases where a focal resistant strain at relatively low frequency is plated together with a larger antibiotic-susceptible population [14, 28]. Other studies (e.g. [36]) have made equivalent measurements without framing the process as stochastic establishment.

To estimate inoculum size, mutant culture is plated alone in a ‘baseline’ environment (benign media), where we assume all cells establish visible colonies. In parallel, culture is plated in ‘test’ environment(s) of interest (e.g. containing antibiotics, and possibly wild-type cells). The probability of establishment of a single mutant cell in test environment *x,* which I denote throughout by *p*_e_ *(x*), is then estimated by:


(1) $$p_{e}(x)=\frac{C_{x}}{C_{0}}$$

where *C_x_* denotes the colony count in environment *x*, and *C_0_* the colony count in the baseline environment. If different dilutions were plated in each environment, the counts should be rescaled appropriately. Note that sampling error in both *C_0_* and *C_x_* contribute to error in estimated *p_e_*.

### Method 2: sector counts in expanding colonies

Establishment has also been studied within radially expanding colonies of fungi and bacteria [11, 13, 17, 29], which present crowded cellular environments for establishing mutants. These studies collectively emphasize how cells’ physical traits, mechanical interactions, mode of growth, and inoculation position influence the strength of genetic drift in spatially structured populations.

In these experiments, labelled mutant and wild-type cultures are mixed, with the mutant at low frequency such that individual lineages can later be resolved. A droplet is inoculated at the centre of an agar plate and incubated to allow colony expansion. Outgrowth is limited by physical space and nutrients, and typically cells must reach and ‘surf’ on the colony front in order to form sizeable descendant populations; hence establishment probability is also called ‘surfing probability’ [11, 29]. Lineages are typically counted as established if they form sectors radiating outward to the colony front, visualized by colour [17] or fluorescence [11, 13, 29]. However, the most appropriate definition of establishment can depend on the study organism’s mode of growth and the application of interest. Work with the mycelially growing fungus *Aspergillus nidulans* quantified both the fraction of mutant spores that ‘escape’ the inoculum area at all, and the subset of those that form sectors [17]. Elsewhere, authors have noted that ‘bubbles’ of mutant cells trapped within the colony could be relevant if they remain viable and could re-grow following clearance of the surrounding wild-type (e.g. by antimicrobial treatment) [37, 38].

Analogous to equation 1, establishment probability in test condition *x* can be estimated by


(2) $$p_{e}(x)=\frac{N_{sec}\left(x\right)}{N_{0}}$$

where *N*_sec_ is the number of mutant sectors (or otherwise defined visible lineages) and *N_0_* is the mutant inoculum size. Most simply, *N_0_* would reflect the total number of mutant cells in the inoculated droplet, estimated from the droplet volume, total cell density and mutant frequency in the inoculating culture. However, inoculation position critically influences establishment, and in some cases, cells starting behind the colony front have negligible chances of establishment [11, 13, 29]. Thus, some studies count only the outermost cell layer in the inoculum, estimating


(3) $$N_{0}=2\pi r_{0}f_{0}$$

where *r_0_* is the radius of the inoculated droplet in units of cell length (hence 2π*r_0_* is the initial number of cells at the colony front), and *f_0_* is the proportion of mutants in the inoculating culture [11, 13, 29].

Finally, if the absolute number of inoculated mutant cells is very small – possibly even zero in some replicates – accounting for variation is critical. The inoculum size can then be described probabilistically with a Poisson distribution [13, 17]. One study explicitly quantified this distribution by counting germinated spores of *A. nidulans* under the microscope, and used it to weight theoretical calculations of *p_e_* into an overall probability of observing *at least one* mutant sector in a colony, which could be directly compared to experimental observations [17]. More generally, an experimental estimate of *p_e_*, accounting for Poisson-distributed inoculum size, could be calculated as described below (equations 6 and 7).

### Method 3: inference from outgrowth in liquid

Establishment can also be studied in liquid culture, in an experiment sometimes called a ‘seeding assay’ [12, 39]. To date, these assays have been conducted with various bacterial species [12, 15, 16, 18, 31, 39], often under antibiotic treatment. Cells are inoculated into liquid media, normally in micro-titre (e.g. 96-well) plates to achieve high replication. Growth is detected by culture turbidity (optical density); when co-culturing the mutant with wild-type cells, mutant presence is determined by its fluorescent signal [12] or by selective plating [16, 18]. An analogous assay, though not phrased in evolutionary terms, has been developed using microfluidic droplets to encapsulate bacteria, with outgrowth detected by total fluorescent signal [40].

A crucial difference from methods 1 and 2 is that detectable growth can be due to one or more established lineages, which cannot be visually distinguished and must be considered statistically. We can first assess the proportion of cultures where *any* mutant(s) establish, based on growth from an arbitrary and unknown inoculum size. ‘Population-scale’ establishment in test condition *x* occurs with estimated probability:


(4) $$P(x)=\frac{n_{e}\left(x\right)}{n_{tot}\left(x\right)}$$

where *n*_tot_ is the total number of replicate cultures, of which *n*_e_ show a detectable mutant population. We expect *n*_e_ to be binomially distributed (with *n*_tot_ trials and probability *P* of ‘success’); thus, standard statistics are applicable for quantifying uncertainty in *P(x*) and significance of differences amongst test conditions. Several studies have addressed stochastic establishment of novel alleles only on this population level [16, 18, 39].

Translating from population-scale (*P*) to per-cell establishment probability (*p*_e_) requires quantification of inoculum size and assumptions about how cells within a culture interact. Existing models assume that each inoculated cell *independently* establishes (or fails to establish) a lineage. This implies that while the overall chance of population growth (*P*) increases with inoculum size, *per-cell* chance (*p*_e_) is independent of inoculum size. This assumption can be checked experimentally [12, 15], and seems reasonable if mutants are at low density, implying interactions with one another are negligible, until establishment is already ‘decided’. This assumption does not preclude interactions with higher-density wild-type cells impacting mutant establishment.

We can then express population-level probability of culture growth, *P*, as the chance that *at least one* inoculated cell independently establishes (each with probability *p*_e_). If inoculum size *N_0_* is known precisely, e.g. by cell sorting [33], then


(5) $$P(x)=1-{\left(1-p_{e}(x)\right)}^{N_{0}}$$

More commonly, however, we inoculate a small volume of highly diluted culture, containing an unknown and random number of cells, shown experimentally to follow a Poisson distribution [12, 18]. Accounting for variable (including possibly zero) inoculum size yields [12, 15, 31]:


(6) $$P(x)=1-exp\left(-{\bar{N}}_{0}p_{e}(x)\right)$$

where ${\bar{N}}_{0}$ is mean inoculum size, which can be estimated by plating [15] or a parallel seeding assay in benign liquid media [12, 31, 39]. In the latter case, we calculate ${\bar{N}}_{0}$ from growth in benign media (*x=0*) using equation 6 with *p_e_(0*)=1, equivalent to the historical ‘most probable number’ method of estimating culture density [41, 42].

Finally, we estimate single-cell establishment probability in test condition *x* by rearranging equation 6 and substituting equation 4 (experimental data) for *P(x*), yielding [12, 15, 31]:


(7) $$p_{e}(x)=\frac{-ln\left(1-P\left(x\right)\right)}{{\bar{N}}_{0}}=\frac{-ln\left(1-n_{e}\left(x\right)/n_{tot}\left(x\right)\right)}{{\bar{N}}_{0}}$$

The above equations give maximum-likelihood point estimates. Confidence intervals on *p*_e_, accounting for uncertainty in both numerator and denominator of equation 7, can be quantified by joint likelihood inference [12] or bootstrapping [15].

## Discussion and outlook

In summary, establishment probability can be estimated in a short experiment with fairly straightforward microbiological techniques and simple mathematical equations. These techniques are broadly applicable: in principle, to any beneficial allele, in any experimentally tractable microbe, in any lab-imposed environment. Existing methods offer alternative advantages: methods 1 and 2 provide direct, intuitive visualization of establishment, whereas method 3 is less intuitive but more amenable to high replication, especially with automated liquid handling. Method 1 requires minimal equipment; method 3 requires a plate reader (potentially capable of fluorescence detection for distinguishing labelled strains); while method 2 often relies on fluorescent microscopy. Different methods also lend themselves more readily to different extensions (discussed below).

Experiments to estimate establishment probability have much in common with fluctuation assays to estimate mutation rate, including their short timescale and explicit consideration of stochasticity, necessitating high replication. Although establishment assays are comparably in their infancy, I argue that they could become just as mainstream as fluctuation assays. Once mutant strains are generated, testing establishment via method 1 or 3 requires similar experimental effort to fluctuation assays, and comparable or simpler calculations. Notably, rigorous estimation of mutation rates surged with the development of accessible software [10, 43–46]. Although calculating point estimates of establishment probability is straightforward, confidence intervals and model validation are more complex. While some custom code is publicly available [12], more user-friendly software could accelerate uptake.

More broadly, establishment assays join a growing suite of techniques to quantify components of microbial adaptation experimentally. Measurements of mutation [8–10] and HGT rates [31], together with establishment probability, parameterize the rate at which adaptive variants arise and escape stochastic loss in the short term. Complementary experiments, e.g. using lineage barcoding [47, 48], can track the frequency dynamics of many competing genotypes over longer timescales.

Establishment of novel beneficial mutants has strong parallels to other stochastic events in ecology and evolution, including colonization of new habitats [33], outgrowth of microbial food contaminants [34], host infection by transmitted pathogens [35, 49, 50], and evolutionary rescue from standing or *de novo* genetic variation [32]. This similarity is reflected mathematically: versions of equations 5 and 6, expressing the probability of a binary outcome (typically growth/detection) due to independent successes of one or more individuals, appear across these contexts, as well as in the ‘P0 method’ of estimating mutation rate [8] or conjugation rate [31]. Analysis tools developed for establishment probability, or vice versa, could thus be applied more broadly.

There is huge scope to expand investigation of stochastic establishment across biological settings. The diversity of microbial life cycles, genetic architecture and gene exchange inspire many possibilities; for instance, recent work compared establishment of alleles on chromosomes versus multicopy plasmids [16]. Another key extension is to test establishment in spatially and temporally varying environments, noting that solid media (methods 1 and 2) versus liquid media (method 3) offer different possibilities. Establishment could also be tested in more complex spatially structured microbial communities by building on method 2. Establishment is particularly sensitive to environmental fluctuations: whereas large populations can buffer declines due to local or temporary poor conditions, rare mutants are vulnerable to extinction. In variable environments, the timing and location of mutant appearance are thus critical to success. Light-inducible mutant phenotypes offer one novel way to test these effects [51].

Another key direction for future work is to unpack the ‘black box’ of cell-level dynamics underlying establishment. Few studies to date [15, 17, 28, 30] have quantitatively compared empirical estimates of establishment probability (as described here) to theoretical predictions. These comparisons could be facilitated by directly visualizing cell population dynamics with time-lapse microscopy, using set-ups on agar (e.g. [30]) or in microfluidic droplets (e.g. [52]) that allow precise enumeration of cells up to sufficiently large population sizes. Single-cell microscopy can also be used to identify factors (including intracellular or micro-environmental) that explain variation in the fate of single cells, e.g. their response to antibiotics [26]. The relative contributions of a large suite of traits can be assessed using machine learning [53]. Future studies could link traits of interest directly to establishment probability.

Measuring establishment probability has important practical implications. Stochastic loss of cells is an unavoidable, but largely overlooked, feature of some common assays. Co-cultured bacterial strains are often distinguished by antibiotic-resistance marker genes and enumerated by selective plating. However, antibiotics can reduce establishment even of ‘resistant’ strains [12, 14, 15, 31], so that only a fraction of cells form colonies (mathematically equivalent to partial plating of the culture). For example, a recent study quantifying conjugation rate found that 99 % of doubly resistant transconjugants failed to form colonies on selective plates, implying conjugation rate would be underestimated without correcting for this effect [31]. The correction factor is not entirely straightforward, because establishment on selective plates occurs independently for each transconjugant cell descending from a single conjugation event. Thus, we cannot simply re-interpret uncorrected estimates as the conjugation rate times the establishment probability. The same issues apply in fluctuation assays, where partial plating (of a specified fraction) can already be handled by some available inference software [10, 45, 46]. However, the establishment probability of spontaneous mutants is not known *a priori* and could only be determined post hoc for a sample of mutants that arose. The impact of unrecognized stochastic establishment on mutation rate estimates from fluctuation assays can be explored through simulations in the Supplementary Material [1].

Another common assay affected by stochastic establishment is the determination of minimum inhibitory concentration (MIC) to quantify a bacterial strain’s susceptibility to an antibiotic. MIC is commonly assessed by visible growth from an inoculum of 5×10^5^ cells ml^−1^ [54], confounding single-cell susceptibility with inoculum size [12, 15, 40]. Assuming cells establish independently, equation 6 predicts that the chance of detecting population growth increases with inoculum size. Although MIC is widely observed to increase with inoculum size [36, 55, 56], data should thus be compared to a null expectation [15] or integrated with additional mechanistic evidence before concluding that cell interactions impact susceptibility. In contrast, directly using establishment probability (e.g. plating efficiency) to quantify antimicrobial susceptibility circumvents this problem: for instance, IC99 (or MIC99) is the concentration reducing establishment (colony counts) by 99 % [27, 57].

Beyond the lab, stochastic establishment has critical implications whenever we aim to promote or hinder microbial adaptation, as in biotechnology or medicine. Environmental manipulations should be designed with establishment as well as selection in mind. For instance, there is strong interest in antimicrobial dosing strategies that limit evolution of resistance, but studies usually focus on identifying concentration ranges or pharmacokinetic measures that avoid selectively favouring resistant subpopulations [5, 58]. Although selection is clearly important, an exclusive focus risks overlooking alternative strategies for driving rare resistant cells extinct. For instance, doses that select for resistance may nonetheless restrict establishment of single cells to low probability [12]. Considering pharmacokinetics, mathematical modelling predicts that fractionating a fixed total dose into frequent small doses achieves greater average population decline, but few large doses can be more effective at eradicating a small partially resistant population [59]. Given these implications, I argue that the stochastic nature of establishment should become a familiar concept amongst applied microbiologists, even where establishment probability is not explicitly quantified.
